# A pilot study on commonality and specificity of copy number variants in schizophrenia and bipolar disorder

**DOI:** 10.1038/tp.2016.96

**Published:** 2016-05-31

**Authors:** J Chen, V D Calhoun, N I Perrone-Bizzozero, G D Pearlson, J Sui, Y Du, J Liu

**Affiliations:** 1 The Mind Research Network, Albuquerque, NM, USA; 2 Department of Electrical Engineering, University of New Mexico, Albuquerque, NM, USA; 3 Departments of Neurosciences and Psychiatry, University of New Mexico School of Medicine, Albuquerque, NM, USA; 4 Olin Neuropsychiatry Research Center, Institute of Living, Hartford, CT, USA; 5 Departments of Psychiatry and Neurobiology, Yale University, New Haven, CT, USA; 6 Brainnetome Center and National Laboratory of Pattern Recognition, Institute of Automation, Chinese Academy of Sciences, Beijing, China

## Abstract

Schizophrenia (SZ) and bipolar disorder (BD) are known to share genetic risks. In this work, we conducted whole-genome scanning to identify cross-disorder and disorder-specific copy number variants (CNVs) for these two disorders. The Database of Genotypes and Phenotypes (dbGaP) data were used for discovery, deriving from 2416 SZ patients, 592 BD patients and 2393 controls of European Ancestry, as well as 998 SZ patients, 121 BD patients and 822 controls of African Ancestry. PennCNV and Birdsuite detected high-confidence CNVs that were aggregated into CNV regions (CNVRs) and compared with the database of genomic variants for confirmation. Then, large (size⩾500 kb) and small common CNVRs (size <500 kb, frequency⩾1%) were examined for their associations with SZ and BD. Particularly for the European Ancestry samples, the dbGaP findings were further evaluated in the Wellcome Trust Case Control Consortium (WTCCC) data set for replication. Previously implicated variants (1q21.1, 15q13.3, 16p11.2 and 22q11.21) were replicated. Some cross-disorder variants were noted to differentially affect SZ and BD, including CNVRs in chromosomal regions encoding immunoglobulins and T-cell receptors that were associated more with SZ, and the 10q11.21 small CNVR (*GPRIN2*) associated more with BD. Disorder-specific CNVRs were also found. The 22q11.21 CNVR (*COMT*) and small CNVRs in 11p15.4 (*TRIM5*) and 15q13.2 (*ARHGAP11B* and *FAN1*) appeared to be SZ-specific. CNVRs in 17q21.2, 9p21.3 and 9q21.13 might be BD-specific. Overall, our primary findings in individual disorders largely echo previous reports. In addition, the comparison between SZ and BD reveals both specific and common risk CNVs. Particularly for the latter, differential involvement is noted, motivating further comparative studies and quantitative models.

## Introduction

Schizophrenia (SZ) and bipolar disorder (BD) are two psychiatric disorders whose diagnostic boundaries remain elusive^1^ and some clinical symptoms can be present in both, including impaired cognitive functions, mood dysregulation and psychosis. Knowledge has accrued, suggesting that this clinical overlap results in part from shared genetic liability.^2, 3^ Both SZ and BD have high heritability estimated to be ~70–80%.^4, 5^ Moreover, it has become clear that both are genetically complex disorders. It is estimated that all common single-nucleotide polymorphisms (SNPs) together explain 20–30% of variation in liability to SZ^6^ and for BD the proportion may reach ~37%.^7^ No estimates are available for aggregated rare structural variants yet. From genome-wide association studies (GWAS), a polygenic SZ component was found to significantly distinguish controls from BD patients, but not patients with six non-psychiatric diseases.^8^ For the variance explained by common SNPs, a high genetic correlation of 0.68 was observed between SZ and BD.^9^

Given quantified coheritability, there is an increasing interest in elucidating the cross-disorder and disorder-specific genetic basis of SZ and BD. A combined data set of five psychiatric disorders identified genome-wide significant SNPs in four regions, including 3p21, 10q24, *CACNA1C* and *CACNB2.*^10, 11^ In contrast, a GWAS in a Swedish population^12^ reported greater involvement of the major histocompatibility complex region in SZ than in BD, consistent with the observation of Ruderfer *et al.*^11^ The miR137 variant, rs1625579, appears to conditionally influence brain function that contributes part of the risk to SZ but not to BD.^13, 14^ Meanwhile, rs9371601 (*SYNE1*), rs10994397 (*ANK3*) and rs12576775 (*ODZ4*) are likely more related to BD risk.^10^

Another line of studies explore associations of copy number variants (CNVs) with SZ or BD. CNVs reflect duplications or deletions of chromosomal segments with lengths greater than one kilobase (kb),^15^ which may result in various downstream effects, including disruptions in gene expression and regulation. The CNV effects can be investigated through overall CNV burden or individual CNVs for their associations with traits of interest (the latter known as GWAS of CNVs). For SZ, a higher burden of rare (population frequency <1%) large (size⩾100 kb) CNVs in cases than controls is documented.^16, 17^ Some rare large variants with high penetrance are also identified, including 1q21.1, 2p16.3, 3q29, 15q13.3, 16p11.2, 17q12 and 22q11.21.^5, 18^ Compared with SZ, the effect of rare large CNVs on BD seems less prominent.^12, 19, 20^ One notion is that this echoes a weaker neurodevelopmental component and less severe cognitive impairments in BD than in SZ.^21^ Moreover, there have been relatively few studies performing GWAS of CNVs in BD. Several rare large CNVs previously implicated in SZ were reported to also contribute to BD risk, including 1q21.1, 3q29, 15q13.3 and 16p11.2.^22, 23^ Meanwhile, no BD-specific CNV has yet been documented.

Considering previous work, we were motivated to conduct a pilot study to investigate the commonality and specificity of CNVs in SZ and BD. We sought to extend this line of research in three directions. First, we conducted unbiased GWAS of CNVs in both SZ and BD, which would enable a comparison to locate cross-disorder and disorder-specific variants. Second, we examined CNVs with a broader spectrum of sizes, as the clinical significance of small CNVs has also been demonstrated in neurodevelopmental disorders.^24^ Last, we examined both rare and common CNVs. Most prior work studied only rare CNVs, which might be attributed to the observation that rare variants are with high penetrance, and common CNVs could be tagged by common SNPs.^25^ To our knowledge, there exist controversies regarding to what extent CNV duplications and non-biallelic CNVs can be tagged by SNPs.^26, 27^ In a more comprehensive investigation on this issue, the Wellcome Trust Case Control Consortium (WTCCC) showed that 79% of the CNVs with frequencies >10% and 22% of the CNVs with frequencies <5% could be tagged by SNPs (*r*^2^>0.8).^28^ On the basis of this, we concluded that a non-negligible portion of CNVs with frequencies >1% could not be effectively tagged by common SNPs and deserve investigation.

## Materials and methods

In this work, we used data provided from the Database of Genotypes and Phenotypes (dbGaP) to evaluate genome-wide CNVs of a wide spectrum of sizes and frequencies for their associations with SZ, or BD or both, and separately for European and African Ancestry (EA and AA) groups. The WTCCC data were employed to validate the dbGaP European Ancestry findings and reduce the risk of false-positives, particularly for small CNVs.^29^

### Genetic data

The sample information is summarized in Supplementary Table S1. The dbGaP data (http://www.ncbi.nlm.nih.gov/gap)^30, 31, 32^ were used as the discovery sample, derived from 2416 SZ patients, 592 BD patients and 2393 controls of EA, as well as 998 SZ patients, 121 BD patients and 822 controls of AA (see supplementary for more details). For all the dbGaP data, DNA was extracted from B Lymphoblastoid Cell Lines transformed by Epstein–Barr virus and genotyping was conducted using Affymetrix SNP Array 6.0. The WTCCC data (https://www.ebi.ac.uk/ega/home)^19, 22, 28, 33^ were used for replication, where the SZ data set (EGAS00000000118) included 2491 controls and 2127 patients, and the BD data set (EGAS00000000001) included 1456 controls and 1845 patients. For both SZ and BD data, DNA was extracted from white blood cells. Regarding genotyping, Affymetrix SNP Array 6.0 was used for the SZ data set, whereas Affymetrix Mapping 500 K was used for the BD data set.

### CNV calls

Stringent quality controls were employed to reduce false-positive findings as much as possible.^30^ In brief, we excluded low-quality samples and potential relatives. Then, Affymetrix Power Tool (www.affymetrix.com/estore/partners_programs/programs/developer/tools/powertools.affx) was used to perform data normalization and extract log R ratio and B allele frequency signals. Samples exhibiting high log R ratio-s.d. (>0.29) were excluded. PennCNV-Affy^34^ was used to generate CNV calls with correction for GC content to avoid spurious CNV calls due to waving effect.^35^ CNVs spanning less than three markers or 1 kb were ignored, as suggested by the PennCNV developer. Meanwhile, Birdsuite^36^ was conducted using the default settings for Affymetrix SNP 6.0. Conservatively, high-confidence CNVs were obtained from those detected by both PennCNV-Affy and Birdsuite and showing overlap ⩾50%. Then, for each analysis group (EA SZ, EA BD, AA SZ and AA BD), sample outliers presenting an excess number of CNVs (>3 s.d.) were further excluded. In the replication step, the same quality control was applied, except that we decided to rely on the conservative PennCNV approach for CNV calling, as the WTCCC genotyping involved the Affymetrix Mapping 500 K array for which Birdsuite is not particularly suited. The resulting CNVs were directly compared with the dbGaP results for confirmation purposes.

### Statistical analyses

We performed association analyses to identify CNVs presenting different frequencies between controls and patients in dbGaP. For each analysis group, we used 500 kb as a size threshold to separate small and large CNVs.^12, 16, 37^ Then, for each category, an iterative process was implemented to aggregate overlapping CNVs into CNV regions (CNVR). Common and rare CNVRs were then determined based on a frequency threshold of 1%. We skipped investigating rare small CNVs as they might bear a high false-positive rate.^24^ The common small, common large and rare large CNVRs were compared against the database of genomic variants (DGV) and we excluded those CNVRs that did not overlap with any DGV-documented CNVR.^30, 38^ This was expected to reduce the possibility of false-positive calls, given that validation with quantitative polymerase chain reaction (qPCR) was not achievable in the current study. Finally, for all the CNVRs entering the association analysis, the copy numbers were categorized into duplication (copy numbers 3 and 4), normal (2) or deletion (0 and 1).

We first examined 15 rare large CNVRs previously implicated in SZ for their associations with both disorders in dbGaP.^18, 22^ A counterpart CNVR was defined based on an overlap⩾50%. One-tailed Fisher’s exact test was used to detect consistent associations with SZ, whereas two-tailed test was employed for BD.^22^ Then, in blind tests, each CNVR was evaluated with analysis of variance for frequency differences between controls and SZ/BD patients. A *P*-value of 0.01 (uncorrected) was used to select out potential important associations, which was a tradeoff for false-negatives, given that associations not reaching genome-wide significance might also be informative.^8^ Meanwhile, each CNVR identified in dbGaP was inspected on the following aspects to guard against false-positives. First, we examined whether it would survive when a more stringent quality control was applied to require each CNV spanning at least 10 markers, which demonstrated a very low false-positive rate through experimental validations.^30^ Second, we examined whether the CNVR showed consistent associations across the experimental batches. Finally, the CNVR was examined in the corresponding WTCCC data where we applied the same procedure to detect small common and large CNVRs. If an overlapping counterpart existed in WTCCC, a consistent association (*P<*0.05) was considered to be a replication for the dbGaP finding.

## Results

All common small, common large and rare large CNVRs identified in dbGaP overlapped with at least one CNVR documented in DGV. The overlap ratio (overlapping base pairs/dbGaP CNVR base pairs) was 0.97±0.11 for common small CNVRs and 0.64±0.33 for all large CNVRs. The average CNV burden was 37.54 CNVs per sample in dbGaP. Using the threshold of 500 kb for size and 1% for frequency, no significant rare large CNV burden was observed in any of the four analysis groups. When a size threshold of 100 kb was used,^16^ a marginal case over-representation (*P*=5.52 × 10^−2^) was noted in EA SZ.

### Fifteen CNV loci previously implicated in SZ

Table 1 shows how the 15 previously implicated CNVRs were associated with SZ or BD in the dbGaP EA data. Some were not captured in the current data. Nevertheless, 1q21.1, 15q13.3, 16p11.2 and 22q11.21 showed consistent SZ associations (*P<*0.05). 3q29 showed a marginal trend (*P*=6.36 × 10^−2^). The 1q21.1 duplication also showed a consistent BD association,^22^ and a significant differential effect was noted between SZ and BD (*P*=0.05). The associations observed from most other CNVRs, although not significant, were consistent with previous reports. Some exceptions included CNVRs in 16p13.11 and 17p12 (SZ) as well as CNVRs in 15q13.3 and 16p11.2 (BD), for which no CNV was observed in the SZ or BD patient group.

### EA small common CNVRs

**Figure 1:**
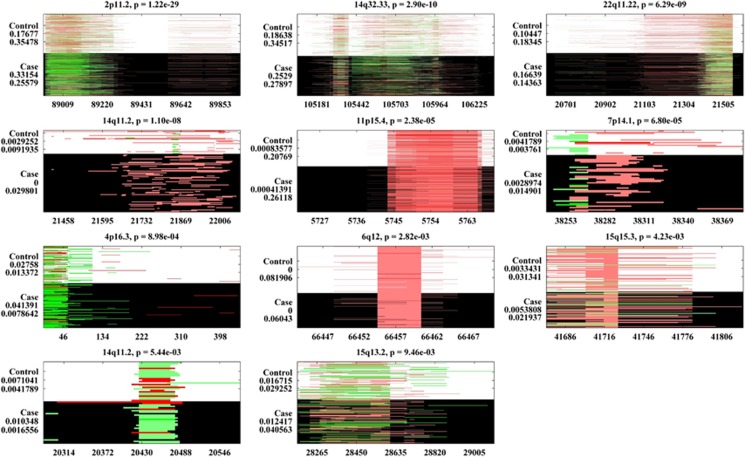
Small (size<500 kb) common (frequency⩾1%) copy number variant regions (CNVRs) associated with schizophrenia (SZ; European Ancestry (EA)). Each subplot represents one identified CNVR. The control group is shown in a background color of white and the case group in black. CNV duplications are plotted in green and deletions in red. The *x* axis displays the CNVs’ positions in the unit of kb. On the *y* axis, ‘Control’ and ‘Case’ groups are marked, each followed by two numbers referring to CNV duplication and deletion frequencies in the specific group.

**Figure 2:**
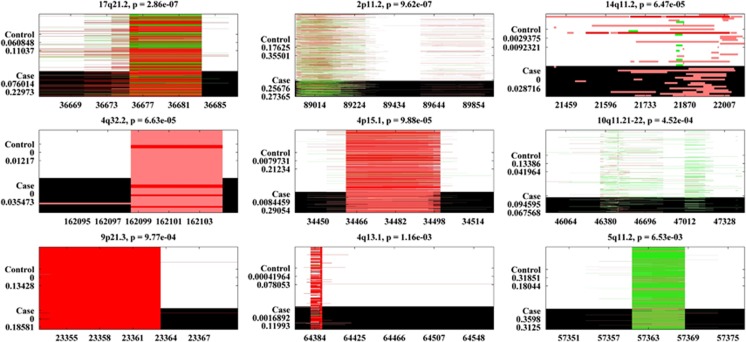
Small (size<500 kb) common (frequency⩾1%) copy number variant regions (CNVRs) associated with bipolar disorder (BD; European Ancestry (EA)). Each subplot represents one identified CNVR. The control group is shown in a background color of white and the case group in black. CNV duplications are plotted in green and deletions in red. The *x* axis displays the CNVs’ positions in the unit of kb. On the *y* axis, ‘Control’ and ‘Case’ groups are marked, each followed by two numbers referring to CNV duplication and deletion frequencies in the specific group.

We identified 367 small common CNVRs in the dbGaP EA SZ data set, 11 of which showed significant associations (*P<*0.01), including 2p11.2, 14q32.33, 22q11.22, 11p15.4, two regions in 14q11.2 and so on (Table 2a and Figure 1). In the dbGaP EA BD data, 9 out of 366 small common CNVRs showed significant associations (Table 2b and Figure 2). Two of these nine CNVRs, 2p11.2 and 14q11.2, showed associations with both SZ and BD. Another CNVR, 10q11.21-22, presented a subthreshold SZ association (*P*=1.11 × 10^−2^). For these three potentially cross-disorder CNVRs, we further tested frequency differences between SZ and BD. Significantly more duplications were observed in SZ than in BD for 2p11.2 (*P*=2.64 × 10^−2^). A marginal group difference was noted for 10q11.21-22 (*P*=5.08 × 10^−2^), with BD patients presenting more deletions. No significant group difference was observed for 14q11.2, although SZ patients showed more deletions. For all the 11 SZ-related dbGaP CNVRs, counterparts were observed in WTCCC, of which six showed significant associations consistent with the dbGaP findings, including 2p11.2, 14q32.33, two regions in 14q11.2 and so on (highlighted in Table 2a). For BD, five out of the nine identified CNVRs had counterparts in WTCCC. None of them showed significant WTCCC associations, although consistent directions of group differences were observed.

### EA large CNVRs

**Figure 3:**
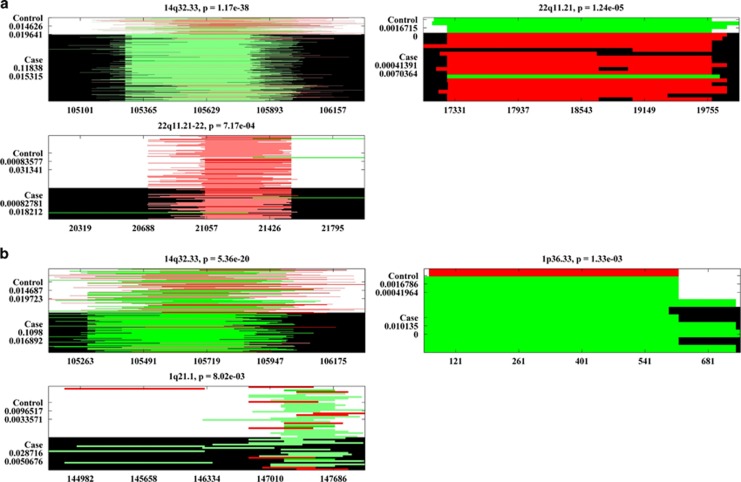
Large (size⩾500 kb) copy number variant regions (CNVRs) associated with schizophrenia (SZ; European Ancestry (EA)) plotted in (**a**) and bipolar disorder (BD; EA) in (**b**). Each subplot represents one identified CNVR. The control group is shown in a background color of white and the case group in black. CNV duplications are plotted in green and deletions in red. The *x* axis displays the CNVs’ positions in the unit of kb. On the *y* axis, ‘Control’ and ‘Case’ groups are marked, each followed by two numbers referring to CNV duplication and deletion frequencies in the specific group.

Overall, 280 large CNVRs were identified in the dbGaP EA SZ data. We skipped 171 singleton CNVs concerning accuracies of statistical tests. Thresholded at *P<*0.01, 14q32.33, 22q11.21 and 22q11.21-22 showed significant SZ associations (Table 2c and Figure 3a). Except for 22q11.21, the other two regions also hosted small common CNVRs and showed consistent associations. In BD, we located 230 large CNVRs, among which 14q32.33, 1p36.33 and 1q21.1 showed significant associations (Table 2d and Figure 3b). A direct comparison suggested a higher (but not significant) frequency of 14q32.33 duplications in SZ than in BD. All the three large CNVRs identified in SZ had counterparts in WTCCC, where 22q11.21 presented a significant SZ association (highlighted in Table 2c). Regarding BD, counterparts were observed in WTCCC for 14q32.33 and 1q21.1; however, neither of them showed consistent and significant associations.

### AA small common CNVRs

We identified 550 and 549 small common CNVRs in the dbGaP AA SZ and BD data, respectively. Ten CNVRs were found to be significantly associated with SZ (Supplementary Table S2a and Supplementary Figure S1). Collectively, 2p11.2, 14q11.2, 7p14.1, 14q32.33 and 22q11.22 showed consistent SZ associations in both EA and AA. For BD, 11 small common CNVRs showed significant associations (Supplementary Table S2b and Supplementary Figure S2). Only the 17q21.2 CNVR was implicated for EA BD association. Besides, 2p11.2 showed a marginal BD association (*P*=1.60 × 10^−2^).

### AA large CNVRs

In the dbGaP AA SZ data, 151 large CNVRs were identified. Only 14q32.33 presented a significant SZ association. Meanwhile, 22q11.21 showed a subthreshold association (*P*=3.90 × 10^−2^, Supplementary Table S2c and Supplementary Figure S3). In BD, 110 large CNVRs were located and the 14q32.33 CNVR again showed a significant association (Supplementary Table S2d and Supplementary Figure S4). Note that this CNVR was consistently identified in all four analysis groups.

All the identified CNVRs showed significant associations when CNVs spanning less than 10 markers were further excluded, except for 9p21.3 (EA BD) where all the CNVs spanned eight markers, which did not appear to be false-positive calls. In addition, all the associations were consistent across batches regarding direction of effect and significance level.

## Discussion

### CNV burden

A marginal rare large CNV burden was observed in EA SZ when the size threshold was 100 kb, consistent with the previous report.^16^ For a threshold of 500 kb, SZ cases showed more rare large CNVs than controls; however, no significant CNV burden was observed, which might be because of the limited sample size, given that variations greater than 500 kb are even rarer.^37^ No significant CNV burden was observed in BD under all conditions, resonating with the common model in the scientific community that CNV burden has a more important role in SZ than in BD risk.^12^ However, this observation awaits further scrutiny, given that the current BD sample is not as well-powered as the SZ sample.^39^

### Fifteen CNV loci previously implicated in SZ

Overall, we observed highly consistent associations (although not all significant) of the 15 CNVRs with SZ or BD in the dbGaP EA data. Four CNVRs showed trends opposite to previous findings, with no CNVs identified in the case group, which could be largely attributed to the limited sample size not being able to capture extremely rare variants (frequency <0.1%). The 1q21.2 duplication was replicated in both SZ and BD, showing a more significant BD association. This differential effect awaits further validations. Another replicated variant, the 15q13.3 deletion, is considered a strong susceptibility factor for SZ;^18^ however, it likely has a role in epilepsy also.^40^

The most significantly replicated finding was the 22q11.21 deletion, that was also identified in the blind test and further validated in the WTCCC data. This CNVR affects multiple genes, among which the most interesting is *COMT*, which has a critical role in the degradative pathway of dopamine and is implicated in various SZ studies.^41, 42^ This CNVR also showed a SZ association in AA (*P*=0.04), suggesting that it confers SZ vulnerability in both populations. In contrast, this CNVR did not show any association with BD. Cautions need to be exercised when interpreting this result. The number of BD cases might not be sufficient to capture this variant, which appears to be extremely rare in BD.^22^ Overall, we speculate that the 22q11.21 deletion is more common and more involved in SZ compared with BD.

### CNVRs in 2p11.2, 7p14.1, 14q32.33, 14q11.2 and 22q11.21-22

These CNVRs are located in regions encoding immunoglobulins and T-cell receptors known to show heterosomic aberrations (chromosomal aberrations in subpopulations of cells).^43, 44^ In general, these regional CNVs detected in DNA from cell lines should be interpreted with caution.^28, 34^ Meanwhile, some studies showed that these CNVs can also be seen in normal B cells, suggesting that the genetic alterations may be B-cell-specific, rather than being introduced as a consequence of Epstein–Barr virus transformation or cell-culturing conditions.^45, 46^

The large 14q32.33 CNVR (affecting *IGHE*, *IGHD* and *IGHM*) showed consistent associations with both SZ and BD for both EA and AA populations, with cases presenting more duplications than controls. These associations were not replicated in WTCCC, likely because of DNA source difference. Echoing this, the 14q32.33 large CNV frequencies differed substantially between dbGaP and WTCCC (Table 2). Collectively, the highly consistent associations suggest that 14q32.33 large CNVR is a cross-disorder variant, which may contribute to SZ and BD risk in a way that the immune system is involved.^47, 48, 49, 50^ The 14q32.33 small CNVR was associated with SZ, but not with BD, in both EA and AA. The SZ association was replicated in WTCCC, although a discrepancy in frequency was again noted. Combining the small and large CNVR data, it appears that the 14q32.33 CNV has a higher frequency in SZ than in BD.

The other CNVRs in 2p11.2, 7p14.1, 14q11.2 and 22q11.22 showed more robust SZ associations than BD. The 2p11.2 small CNVR (*IGK*) exhibited significant SZ and BD associations; yet only the SZ association was replicated in WTCCC. The direct SZ versus BD comparison confirmed a significant group difference (*P*=2.64 × 10^−2^). The 7p14.1 small CNVR (*TRG*) showed a more significant SZ association than BD. The 14q11.2 small CNVR’s SZ association, but not BD, was replicated in WTCCC. Both small and large 22q11.21-22 CNVRs showed associations only with SZ.

Genetic variants in the constant region of immunoglobulin gamma chains (located in 14q32) are suggested as modifying certain immunoevasion strategies of herpes simplex virus type 1 and human cytomegalovirus, which are possibly implicated in SZ-related cognitive impairment.^51^ T cells have an important role in the adaptive immune system responsible for recognizing antigens bound to major histocompatibility complex molecules,^52^ whose SNPs have been identified as promising risk factors in GWAS of SZ, but not BD.^14, 53^ Indeed, differential involvement of major histocompatibility complex region^12, 54^ and differential regulation of the innate immune response^55^ between SZ and BD were both noted. Overall, our observations echo these previous findings in that CNVRs affecting immunoglobulins and T-cell receptors showed more robust SZ associations than BD. However, further comparative studies are needed to confirm the differential involvement of the corresponding immune system in SZ and BD.

### Small CNVR in 10q11.21-22

This CNVR showed a more significant BD association than SZ, and a differential frequency (*P*=5.08 × 10^−2^) was noted between SZ and BD. However, the significant EA BD association was not replicated in WTCCC, likely because of the genotyping array difference. This CNVR affects some important genes, including *GPRIN2* involved in the control of neurite outgrowth.^56^ In addition, this region was highlighted in a meta-analysis of 18 BD genome data with the most significant evidence for BD linkage.^57^ Overall, our results echo previous work, in that this CNVR might be a more important BD risk factor.

### Small CNVRs in 11p15.4 and 15q13.2

These two small CNVRs showed significant associations with SZ only, suggesting SZ specificity. The 11p15.4-affected genes include *TRIM5* and *TRIM22*, known as intrinsic immune factors against retroviruses and implicated in the etiology of multiple sclerosis.^58^ Interestingly, a genetic pleiotropy was observed between multiple sclerosis and SZ but not BD,^54^ which coincides with our observation that 11p15.4 is associated with only SZ. This CNVR deserves further investigation for its contribution to SZ, which might help better differentiate the disorder from BD. Deletions in 15q13.2-13.3 have been implicated for SZ risk.^59, 60^ The disrupted genes include *TRPM1*, *CHRFAM7A*, *MTMR10* and *MTMR15,* which are involved in DNA repair^60, 61^ and various neuropsychiatric disorders, including schizophrenia and addiction.^62, 63, 64, 65^ Overall, the structural variant in 15q13.2-13.3 is likely a risk factor for SZ.

### Small CNVRs in 17q21.2, 9p21.3 and 9q21.13

These three CNVRs showed BD associations only. A meta-analysis of 18 BD genome scan highlighted all these three regions for top BD linkage,^57^ whereas none of them showed up in a companion meta-analysis of SZ, echoing our findings regarding BD specificity.^66^ However, the question remains as to what functional consequences these variants might exert.

### Other CNVRs

Other CNVRs presented SZ and BD associations in the current work. However, the association was either observed in a single analysis group, or not replicated in the WTCCC data. These data should be treated with caution, although some were implicated in previous studies. For instance, the 1q21.1 large CNVR affects gene *PRKAB2*, which is implicated in various neuropsychiatric conditions.^67^ The 15q11.2 small CNVR is in the Prader–Willi region close to the rare 15q11.2 deletion known for SZ association.^33, 68^ The 8p23.2 affects gene *MCPH1* having a role in neurogenesis.^69^ Overall, these findings carry potential information of interest, but require further confirmatory evidence.

### Common CNVRs tagged by SNPs?

We calculated the correlations between the 41 identified common CNVRs (Table 2) and neighboring common SNPs within a window of 2 Mb. Eleven CNVRs were tagged by neighboring SNPs with *r*^2^>0.2.^14^ Fourteen CNVRs exhibited higher correlations with distant SNPs (>2 Mb) than neighboring SNPs. The remaining 16 CNVRs showed *r*^2^ of 0.08±0.05. Whereas the accuracies of CNV–SNP correlations are limited to the current sample sizes and genotyping arrays, the results suggest that a portion of common CNVRs cannot be tagged by common SNPs.

The current study needs to be interpreted in light of several limitations. First, the identified CNVs were not validated with qPCR because of the unavailability of DNA samples. Instead, we employed stringent quality control to avoid false-positive findings as much as possible. In the discussion, we focused more on replicated results or those identified in more than one analysis group, which were more likely true positives. Other findings were considered more preliminary and require future verification. Second, the DNA samples of dbGaP were obtained from Epstein–Barr virus-transformed B lymphoblastoid cell lines; therefore, the regions encoding immunoglobulins might show heterosomic aberrations.^43^ In the current study, we observed frequency differences for CNVs in 2p11.2, 14q32.33 and 22q11.22 between dbGaP and WTCCC, whereas no dramatic frequency differences for CNVs in 14q11.2 and 7p14.1 (encoding T-cell receptors). We speculate that the former CNVs might be specific to B cells;^46^ however, this could not be verified at present. Nevertheless, this should not substantially compromise the observed CNV associations. In dbGaP, these CNVRs showed highly consistent SZ and BD associations in EA and AA, which were not likely artifacts, but reflected true group differences. Last, the BD association analyses were underpowered and the WTCCC replication was affected by the genotyping array difference. Consequently, we may have missed a number of BD variants.

In brief, we conducted a pilot study to examine the commonality and specificity of small common and large CNVs in SZ and BD. On the basis of the results, the following conclusions can be drawn. For SZ, there is a large CNV burden effect. The CNV burden for BD is less conclusive. CNVs in regions encoding immunoglobulins and T-cell receptors are associated with both SZ and BD, but may have a more important role in SZ. One speculation is that this reflects differential involvement of the immune system. In contrast, the 10q11.21-22 variant affecting *GPRIN2* contributes more to BD risk. The 22q11.21 variant affecting *COMT*, and variants in 11p15.4 and 15q13.2, are likely SZ-specific, with no BD associations observed. The counterpart is variants in 17q21.2, 9p21.3 and 9q21.13, which only show BD associations. Overall, our primary findings in each disorder are largely consistent with previous reports. The comparison between SZ and BD findings reveals both specific and common risk CNVs. For the latter, differential involvement is noted, motivating further comparative studies and quantitative models.

## Figures and Tables

**Table 1:** SZ and BD associations of 15 previously implicated large CNV loci (EA)

*(a) SZ associations of 15 previously implicated CNV loci (dbGaP EA)*					
*CNVR*	*Region start*	*Region end*	*HC freq*	*SZ freq*	P*-value*^a^
1q21.1 dup	144 643 825^b^	146 395 960	0.00000	0.00210	3.19E−02
1q21.1 del	144 643 825	146 395 960	0.00042	0.00083	5.04E−01
*NRXN1* del	50 429 732	51 543 819	0.00000	0.00083	2.52E−01
3q29 del	197 190 376	198 838 385	0.00000	0.00166	6.36E−02
WBS dup	72 297 543	73 780 040	0.00000	0.00124	1.27E−01
*VIPR2* dup	158 137 395	158 819 765	0.00042	0.00083	5.04E−01
15q11.2 del	20 302 458	20 852 214	0.00084	0.00124	5.05E−01
AS/PWS dup	20 224 763	26 742 083	0.00000	0.00041	5.02E−01
15q13.3 del	28 173 703	30 664 276	0.00042	0.00290	3.61E−02
16p13.11 dup	15 306 385	16 588 399	0.00042	0.00000	1.00E+00
16p11.2 del	—	—	—	—	—
16p11.2 dup	29 158 416	30 134 444	0.00000	0.00373	2.02E−03
17p12 del	14 023 683	15 425 596	0.00042	0.00000	1.00E+00
17q12 del	31 610 407	33 552 901	0.00000	0.00041	5.02E−01
22q11.2 del	17 028 880	20 058 138	0.00000	0.00662	1.61E−05

*(b) BD associations of 15 previously implicated CNV loci (dbGaP EA)*					
*CNVR*	*Region start*	*Region end*	*HC freq*	*BD freq*	P*-value*^c^
1q21.1 dup	144 643 825	146 395 960	0.00000	0.00507	7.85E−03
1q21.1 del	144 643 825	146 395 960	0.00042	0.00000	1.00E+00
*NRXN1* del	—	—	—	—	—
3q29 del	—	—	—	—	—
WBS dup	—	—	—	—	—
*VIPR2* dup	158 137 395	158 652 131	0.00042	0.00000	1.00E+00
15q11.2 del	20 302 458	20 852 214	0.00084	0.00000	1.00E+00
AS/PWS dup	—	—	—	—	—
15q13.3 del	28 173 703	30 337 043	0.00042	0.00000	1.00E+00
16p13.11 dup	15 306 385	16 588 399	0.00042	0.00000	1.00E+00
16p11.2 del	—	—	—	—	—
16p11.2 dup	29 425 212	30 099 408	0.00084	0.00000	1.00E+00
17p12 del	14 019 001	15 467 881	0.00042	0.00169	3.58E−01
17q12 del	—	—	—	—	—
22q11.21 del	17 052 885	20 058 138	0.00000	0.00169	1.99E−01

Abbreviations: BD, bipolar disorder; CNV, copy number variant; CNVR, CNV region; dbGaP, Database of Genotypes and Phenotypes; EA, European Ancestry; freq, frequency; HC, healthy control; SZ, schizophrenia.

a Fisher exact test, one-tailed (Rees *et al.*^18^).

b Positions are in bp for UCSC Build hg18.

c Fisher exact test, two-tailed (Green *et al.*^22^).

**Table 2:** SZ and BD associations of 15 previously implicated rare large CNV loci (EA)

*CNVR*	*dbGaP*				*WTCCC*				*Genes*
	*Region start*^a^	*Region end*	*CNV freq*	P*-value*	*Region start*	*Region end*	*CNV freq*	P*-value*	
*(a) Small common CNVRs significantly associated with SZ (EA)*									
** 2p11.2**^b^	**88 905 263**^c^	**89 958 702**	**0.55683**	**1.22E−29**	**88 942 380**	**89 958 702**	**0.05760**	**1.39E−02**	***IGK***
** 14q32.33**	**105 051 752**	**106 340 497**	**0.53083**	**2.90E−10**	**106 162 138**	**106 282 826**	**0.03833**	**3.19E−05**	***MIR4507, CRIP2, IGHG1, IGHE, IGHD, IGHM***
** **22q11.22	20 602 229	21 605 367	0.29867	6.29E**−**09	21 550 094	21 605 367	0.00173	1.13E**−**01	*IGL1, GGTLC2, PPM1F, PRAME, TOP3B, VPREB1, ZNF280A, ZNF280B*
** 14q11.2**	**21 389 110**	**22** **076 067**	**0.02056**	**1.10E−08**	**21** **566** **254**	**22** **137 883**	**0.03811**	**4.75E−06**	***TRA***
** 11p15.4**	**5** **722 264**	**5** **768 936**	**0.23539**	**2.38E−05**	**5 733 116**	**5 774 897**	**0.38263**	**1.67E−05**	***OR52N1, OR52N5, TRIM5, TRIM22***
** **7p14.1	38 239 855	38 384 552	0.01249	6.80E−05	38 183 237	38 384 552	0.03573	2.74E−01	*TARP, TRG*
** **4p16.3	2281	442 084	0.04474	8.98E−04	2281	310 589	0.06735	7.86E−01	*ZNF595*
** **6q12	66 444 740	66 470 544	0.06993	2.82E−03	66 436 632	66 508 278	0.08770	2.79E−01	*EYS*
** **15q15.3	41 672 410	41 821 698	0.03043	4.23E−03	41 632 714	41 801 547	0.02923	3.30E−01	*CATSPER2, CKMT1A, CKMT1B, STRC*
** 14q11.2**	**20 284 485**	**20 576 165**	**0.01189**	**5.44E−03**	**20 415 547**	**20 495 188**	**0.01602**	**1.62E−04**	***FAM12A, FAM12B, METT11D1, SLC39A2, NDRG2, RNASE1, TPPP2***
** 15q13.2-13.3**	**28 173 703**	**29 097 455**	**0.04978**	**9.46E−03**	**28 173 703**	**28 875 769**	**0.08835**	**4.50E−02**	***ARHGAP11B, TRPM1, CHRFAM7A, MTMR10, MTMR15,***
** **10q11.21-22^d^	45 905 767	47 525 233	0.16183	1.11E−02	45 613 625	47 565 585	0.26180	1.42E−01	*GPRIN2, SYT15, NPY4R, ANXA8*

*(b) Small common CNVRs significantly associated with BD (EA)*									
17q21.2	36 666 936	36 687 067	0.19798	2.86E−07	—	—	—	—	*KRTAP9-6*
2p11.2	88 909 234	89 958 702	0.53109	9.62E−07	89 066 885	89 912 849	0.04393	1.60E−01	*IGK*
14q11.2	21 389 110	22 076 067	0.01546	6.47E−05	21 697 688	22 170 749	0.01181	1.40E−01	*TRA, TRAC*
4q32.2	162 093 356	162 104 799	0.01681	6.63E−05	162 084 190	162 365 231	0.01333	5.96E−01	Intergenic
4p15.1	34 441 990	34 522 011	0.23597	9.88E−05	—	—	—	—	Intergenic
10q11.21-22	45 905 767	47 468 066	0.17311	4.52E−04	47 030 119	47 485 249	0.03302	3.99E−01	*GPRIN2, SYT15, NPY4R, ANXA8*
9p21.3	23 353 115	23 369 719	0.14454	9.77E−04	—	—	—	—	Intergenic
4q13.1	64 364 107	64 567 234	0.08706	1.16E−03	64 353 835	65 004 045	0.00030	2.60E−01	Intergenic
5q11.2	57 348 992	57 377 909	0.53345	6.53E−03	—	—	—	—	Intergenic
									
*(c) Large CNVRs significantly associated with SZ (EA)*									
14q32.33	104 969 537	106 288 935	0.08283	1.17E−38	105 413 362	106 031 276	0.00022	2.79E−01	*C14orf80, CRIP1, CRIP2, MTA1, TMEM121, IGHM, IGHD, IGHE, IGHG1, FAM30A, ADAM6*
**22q11.21**	**17 028 880**	**20 058 138**	**0.00443**	**1.24E−05**	**17 112 919**	**20 798 619**	**0.00325**	**3.86E−02**	***DGCR2, HIRA, PRODH, COMT, SNAP29***
22q11.21-22	20 134 576	21 980 433	0.02600	7.17E−04	21 327 811	23 394 964	0.00065	6.58E−01	*IGL1, GGTLC2, PPM1F, PRAME, TOP3B, VPREB1, ZNF280A, ZNF280B, MAPK1, BCR, GNAZ*
									
*(d) Large CNVRs significantly associated with BD (EA)*									
14q32.33	105 149 735	106 288 935	0.05277	5.36E−20	105 149 735	106 011 769	0.00182	7.77E−03	*IGHM, IGHD, IGHE, IGHG1, FAM30A, ADAM6*
1p36.33	51 598	751 981	0.00370	1.33E−03	—	—	—	—	*OR4F5*
1q21.1	144 643 825	148 024 665	0.01714	8.02E−03	144 106 961	144 943 150	0.00061	2.09E−01	*PRKAB2, CHD1L, BCL9, FCGR1B*

Abbreviations: BD, bipolar disorder; CNV, copy number variant; CNVR, CNV region; dbGaP, Database of Genotypes and Phenotypes; EA, European Ancestry; freq, frequency; SZ, schizophrenia; WTCC, Wellcome Trust Case Control Consortium.

a In all the tables, region start and end reflect the overall CNVR boundary, which is determined based on all the overlapping CNVs.

b The CNVRs replicated in the WTCCC data are highlighted in bold.

c Positions are in bp for UCSC Build hg18.

d Promising region, although showing a subthreshold *P-*value.
