# Supplementary material for: A pilot study on commonality and specificity of copy number variants in schizophrenia and bipolar disorder
Source: Transl Psychiatry. 2016 May 31;6(5):e824–. doi: 10.1038/tp.2016.96 (PMC5545651; doi:10.1038/tp.2016.96)
Supplement: Supplementary Information [file tp201696x1.docx]

***Genetic data***

The dbGaP data (http://www.ncbi.nlm.nih.gov/gap) were used as the discovery sample. For the EA group, the SZ analysis included 2,393 controls and 2,416 patients from the Genome-Wide Association Study of Schizophrenia (GAIN, dbGaP phs000021.v3.p2) and Molecular Genetics of Schizophrenia - nonGAIN Sample (MGS_nonGAIN, dbGaP phs000167.v1.p1). For the EA BD analysis, 2,383 controls and 592 patients were aggregated from GAIN, MGS_nonGAIN and the Whole Genome Association Study of Bipolar Disorder (dbGaP phs000017.v3.p1). The AA sample was smaller than the EA, with the SZ analysis including 822 controls and 998 patients from GAIN and MGS_nonGAIN, and the BD analysis including 819 controls and 121 patients from GAIN, MGS_nonGAIN and Whole Genome Association Study of Bipolar Disorder. Table S1 lists the sample information. Note that controls were largely shared between the SZ and BD analyses for both EA and AA data. The slight differences were due to outlier detection in individual analysis groups (EA SZ, EA BD, AA SZ and AA BD). For all the dbGaP data, DNA was extracted from B Lymphoblastoid Cell Lines (B-LCLs) transformed by Epstein–Barr virus (EBV) and genotyping was conducted using Affymetrix SNP Array 6.0.

**CNV calls**

In the discovery step, two different algorithms, PennCNV-Affy and Birdsuite were used to detect CNVs and generate CNV calls from the raw dbGaP data. Stringent quality controls were employed to reduce false positive findings as much as possible. First we excluded samples that failed the standard SNP quality controls as provided from the original studies, including sex-check failure, low SNP genotyping rate (>0.95) and high heterozygosity (>3SD). For duplicated samples and potential relatives with identity-by-descent>0.1875, only one sample was retained. Then Affymetrix Power Tool (www.affymetrix.com/estore/partners_programs/programs/developer/tools/powertools.affx) was used to perform data normalization and extract log R ratio (LRR) and B allele frequency (BAF) signals from raw CEL files, with the options of “median-smoothing” and “quantile-normalization”. The LRR and BAF information was then used by PennCNV-Affy to generate autosomal CNV calls based on a trained hidden Markov model. Particularly, a correction for GC-content was integrated into PennCNV-Affy to mitigate the waving effect due to hybridization bias of DNA quantities ([39](#_ENREF_39)). Based on the PennCNV-Affy reports, samples exhibiting high LRR-SD (>0.29) were further excluded to avoid spurious CNV calls due to relatively strong waving effects. For the remaining samples, CNVs spanning less than three markers or 1Kb were ignored. And for each analysis group (EA SZ, EA BD, AA SZ and AA BD), sample outliers presenting an excess number of CNVs (>3SD) were also excluded, as suggested by the PennCNV developer. Meanwhile, Birdsuite was conducted using the default settings for Affymetrix SNP 6.0. Then conservatively, a CNV detected by PennCNV-Affy was considered as “high confidence” only when it was consistently detected by Birdsuite and showed overlap≥50%. For the high confidence CNVs, the boundaries and copy numbers were kept consistent with the PennCNV-Affy results. In the replication step, as the genotyping involved the Affymetrix Mapping 500K array for which Birdsuite is not particularly suited, we decided to rely on the conservative PennCNV-Affy approach for CNV calling in the WTCCC data and directly compared the resulting CNVs with the dbGaP results for confirmation purposes.

**Table S1: Sample information.**

|  | **dbGaP (EA)** | **dbGaP (AA)** | **WTCCC (EA)** |
| --- | --- | --- | --- |
| **SZ** | 2393 HC (1167 male, 1225 female, 1 NA^a^) | 822 HC (331 male, 490 female, 1 NA) | 2491 HC (1244 male, 1247 female, 0 NA) |
|  | 2416 SZ (1686 male, 728 female, 2 NA) | 998 SZ (629 male, 361 female, 8 NA) | 2127 SZ (1323 male, 804 female, 0 NA) |
|  | 153 NA (87 male, 66 female, 0 NA) | 60 NA (31 male, 29 female, 0 NA) | 0 NA |
| **BD** | 2383 HC (1164 male, 1218 female, 1 NA) | 819 HC (329 male, 489 female, 1 NA) | 1456 HC (703 male, 753 female, 0 NA) |
|  | 592 BD (299 male, 287 female, 6 NA) | 121 BD (31 male, 88 female, 2 NA) | 1845 BD (687 male, 1158 female, 0 NA) |
|  | 0 NA | 0 NA | 0 NA |

^a^Subjects without self-reported sex information (NA) were excluded from the analysis due to sex check not being conducted.

**Table S2: CNVRs associated with SZ or BD (AA).**

| **Table S2a: Small common CNVRs significantly associated with SZ (dbGaP AA)** | | | | | |
| --- | --- | --- | --- | --- | --- |
| CNVR | Region start | Region end | CNV freq | p-value | Genes |
| 2p11.2 | 88,523,415 | 89,958,702 | 0.54521 | 8.92E-10 | C2orf51, EIF2AK3, RPIA, IGK, |
| 14q11.2 | 21,622,842 | 22,058,411 | 0.03032 | 4.51E-09 | TRA |
| 7p14.1 | 38,250,268 | 38,351,366 | 0.01809 | 6.65E-07 | TARP, TRG |
| 14q32.33 | 105,100,682 | 106,356,482 | 0.53351 | 6.17E-06 | IGHM, IGHD, IGHE, IGHG1, FAM30A, ADAM6 |
| 22q11.22 | 20,637,519 | 21,589,859 | 0.28564 | 1.18E-04 | IGL1, GGTLC2, PPM1F, PRAME, TOP3B, VPREB1, ZNF280A, ZNF280B |
| 2p25.3 | 4,183,562 | 4,214,680 | 0.05798 | 2.50E-03 | intergenic |
| 15q11.2 | 21,646,501 | 22,359,729 | 0.22234 | 3.27E-03 | PWRN2, PWRN4 |
| 10q21.1 | 54,789,031 | 54,812,415 | 0.01702 | 3.62E-03 | intergenic |
| 8p11.23 | 39,344,897 | 39,515,014 | 0.29096 | 7.96E-03 | ADAM5P, ADAM3A |
| 4q13.3 | 73,634,063 | 73,648,872 | 0.05851 | 9.58E-03 | ADAMTS3 |
| **Table S2b: Small common CNVRs significantly associated with BD (dbGaP AA).** | | | | | |
| CNVR | Region start | Region end | CNV freq | p-value | Genes |
| 1p21.3 | 94,903,266 | 94,928,185 | 0.05851 | 6.31E-04 | LINC01057 |
| 17q21.2 | 36,666,936 | 36,687,067 | 0.23511 | 8.58E-04 | KRTAP9-6 |
| 3p22.2 | 37,952,421 | 37,962,146 | 0.03936 | 1.29E-03 | CTDSPL |
| 1q23.1 | 156,781,568 | 156,790,295 | 0.05532 | 1.30E-03 | OR6Y1 |
| 8p23.2 | 6,090,256 | 6,290,919 | 0.01383 | 1.89E-03 | MCPH1 |
| 2q14.1 | 115,112,891 | 115,120,548 | 0.04149 | 2.26E-03 | DPP10 |
| 7p11.2 | 54,152,597 | 54,165,050 | 0.03298 | 2.40E-03 | Intergenic |
| 9q21.13 | 78,955,072 | 78,980,675 | 0.01383 | 3.97E-03 | Intergenic |
| 5p15.32 | 5,731,997 | 5,734,033 | 0.02340 | 5.74E-03 | Intergenic |
| 2p16.2 | 52,744,917 | 53,075,671 | 0.01064 | 8.56E-03 | MIR4431 |
| 1p36.11 | 25,447,058 | 25,553,370 | 0.09681 | 9.12E-03 | RHD, TMEM50A,  AX747205 |
| 2p11.2^a^ | 88,523,415 | 89,408,107 | 0.49681 | 1.60E-02 | C2orf51, EIF2AK3,  RPIA, IGK |
| **Table S2c: Large CNVRs significantly associated with SZ (dbGaP AA).** | | | | | |
| CNVR | Region start | Region end | CNV freq | p-value | Genes |
| 14q32.33 | 105,071,276 | 106,269,389 | 0.11755 | 2.10E-20 | IGHM, IGHD, IGHE, IGHG1, FAM30A, ADAM6 |
| 22q11.21 | 17,256,428 | 19,925,178 | 0.00213 | 3.90E-02 | DGCR2, HIRA, PRODH, COMT, SNAP29 |
| **Table S2d: Large CNVRs significantly associated with BD (dbGaP AA).** | | | | | |
| CNVR | Region start | Region end | CNV freq | p-value | Genes |
| 14q32.33 | 105,079,225 | 106,269,389 | 0.06277 | 1.78E-08 | IGHM, IGHD, IGHE, IGHG1, FAM30A, ADAM6 |

^a^Promising region though showing a sub-threshold p-value.


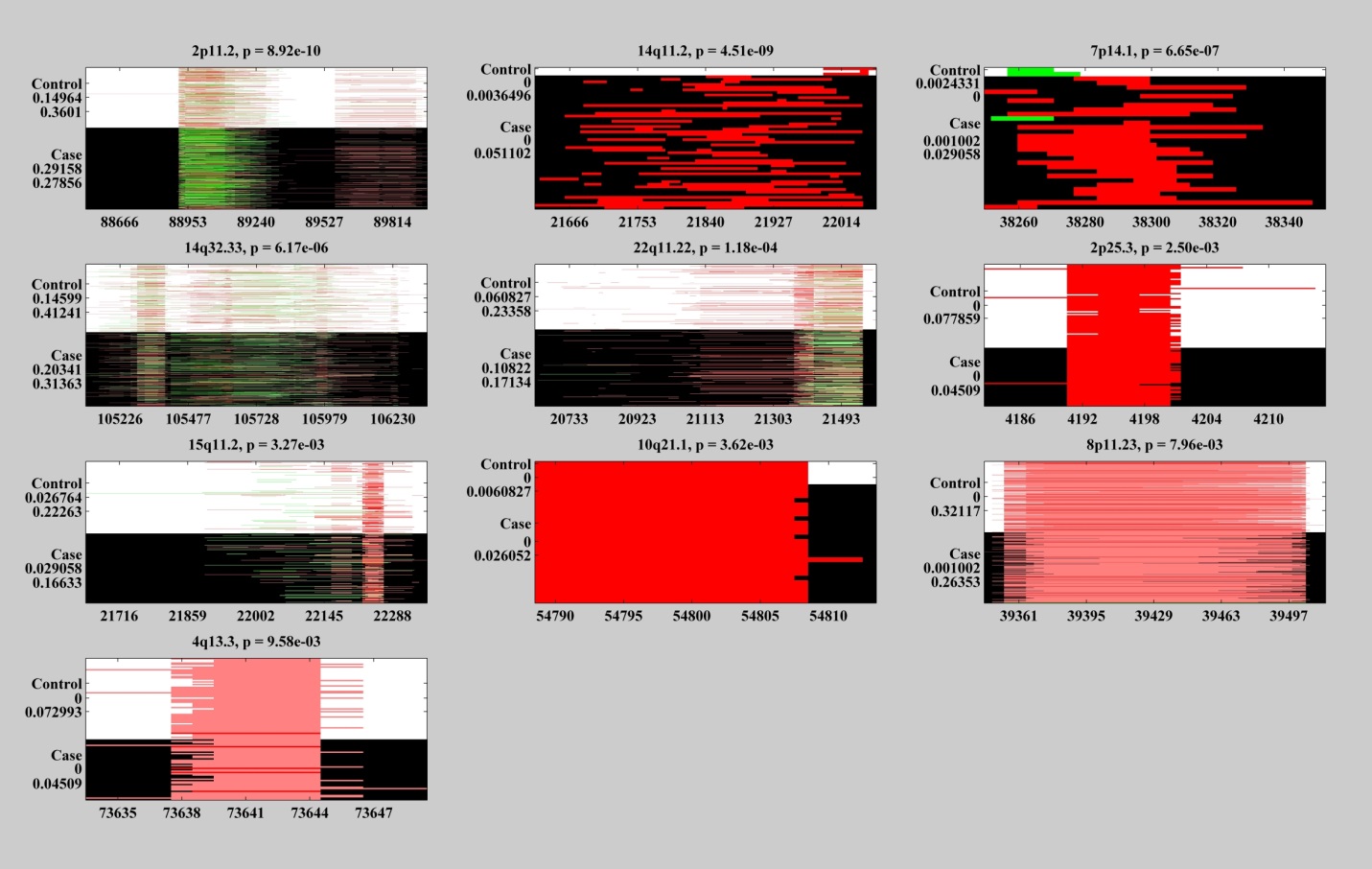


Figure S1: Small (size<500Kb) common (frequency≥1%) CNVRs associated with SZ (AA).


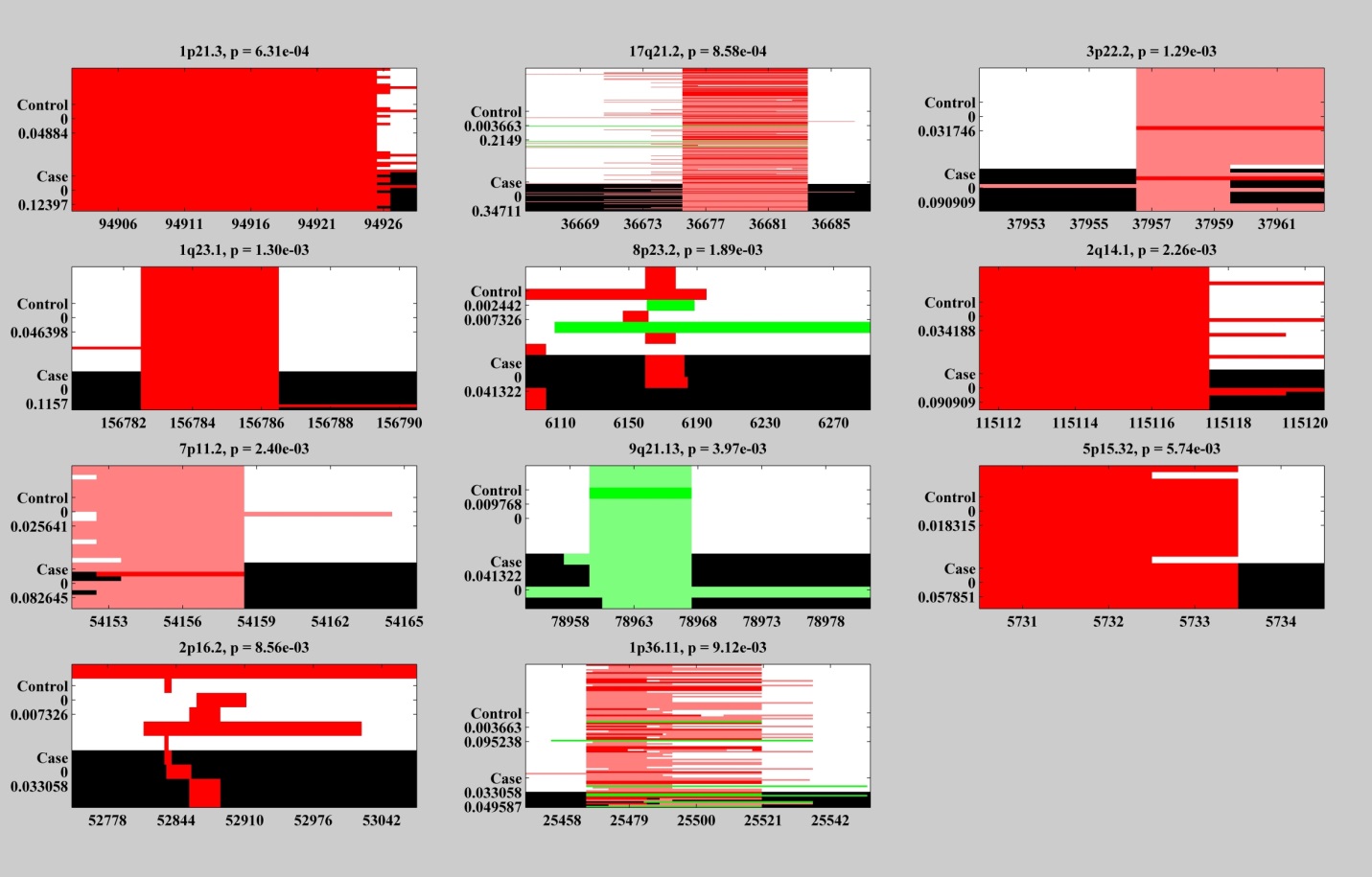


Figure S2: Small (size<500Kb) common (frequency≥1%) CNVRs associated with BD (AA).


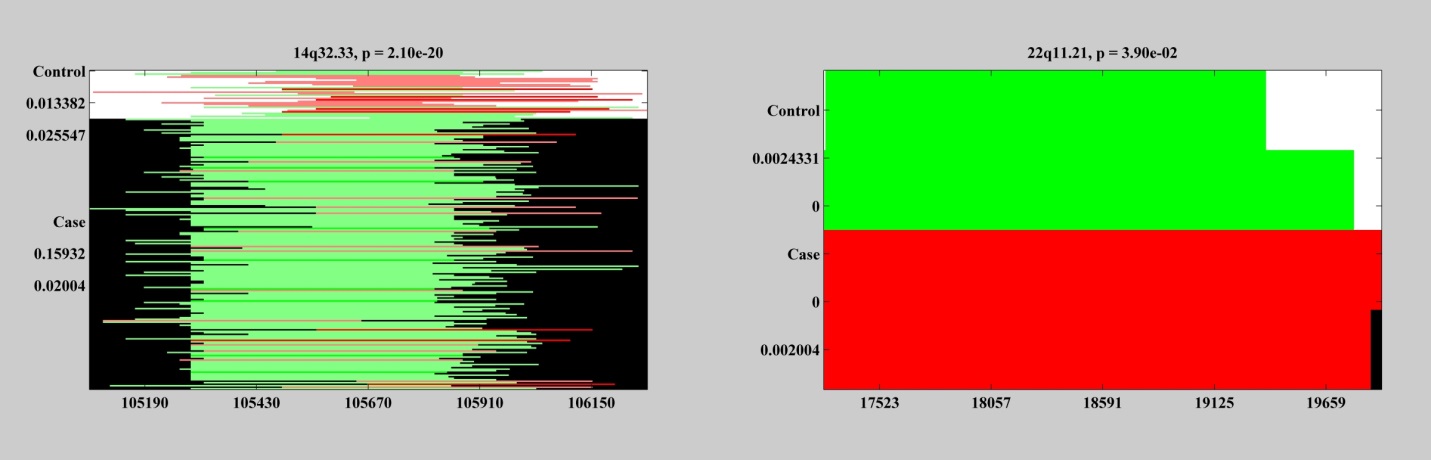


Figure S3: Large (size≥500Kb) CNVRs associated with SZ (AA).


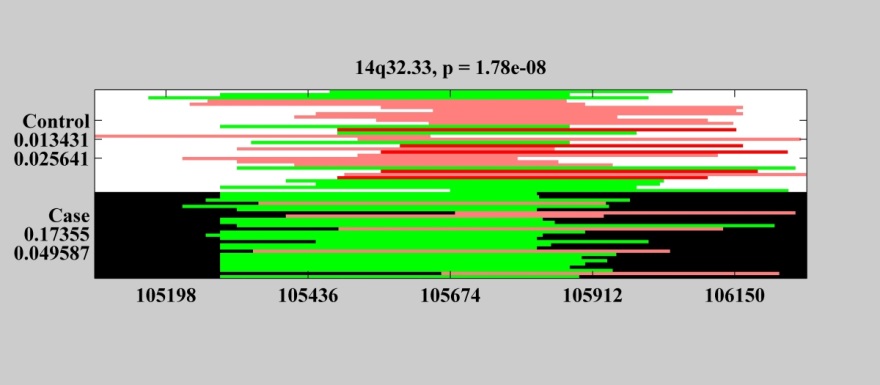


Figure S4: Large (size≥500Kb) CNVRs associated with BD (AA).
